# Aortic Pulse Wave Velocity and Extracellular Water Expansion in Hemodialysis Patients

**DOI:** 10.1111/aor.15015

**Published:** 2025-05-07

**Authors:** Roohi Chhabra, Andrew Davenport

**Affiliations:** ^1^ UCL Centre for Kidney & Bladder Health, Royal Free Hospital University College London London UK

**Keywords:** bioimpedance, hemodialysis, hypertension, mortality, pulse wave velocity

## Abstract

**Background:**

Pulse wave velocity (PWV), a measurement of arterial stiffness, is a risk factor for cardiovascular mortality in hemodialysis patients. Debate continues as to whether PWV is increased by volume overload. As such, we reviewed the association between volume overload and PWV.

**Methods:**

We measured aortic PWV (aPWV) in hemodialysis patients attending routine dialysis sessions with contemporaneous measurements of extracellular water/total body water (ECW/TBW) ratios using multifrequency bioimpedance.

**Results:**

A total of 102 patients, 63.7% male, mean age 63.5 ± 15.9 years, 44.1% diabetic, median dialysis duration 22.2 (4.9–52.8) months, weight 73.7 ± 15.8 kg, and central systolic blood pressure 157 ± 35 mmHg had a mean aPWV of 10.1 ± 2.5 m/s. Patients dialyzed with a low dialysate calcium (median 1.0 [1.0–1.25] mmol/L). Patients with aPWV of ≥ 10 m/s were older (72.9 ± 10.3 vs. 52.6 ± 14.4 years, *p* < 0.01) with a higher ECW/TBW ratio (40.6 ± 1.3 vs. 39.6 ± 1.7, *p* < 0.001), with no differences in active vitamin D_3_ or calcium‐containing medications. Aortic PWV was associated with age (*r* = 0.9, *p* < 0.001) and ECW/TBW (*r* = 0.33, *p* = < 0.001), and after adjusting aPWV for age, ECW/TBW remained higher (40.5 ± 1.5 vs. 39.5 ± 1.5, *p* < 0.001). On multivariable testing, ECW/TBW remained independently associated with a raised aPWV (odds ratio [OR] 1.59 (95% confidence intervals [CI] 1.06–2.41), *p* = 0.026), and after age adjustment (OR 1.74 (95% CI 1.19–2.53, *p* = 0.004)).

**Conclusions:**

This study highlights the association between volume overload, as determined by bioimpedance in hemodialysis patients, and arterial stiffness measured by aPWV. These results would reinforce the importance of improving volume control in hemodialysis patients to reduce cardiovascular risk.

## Graphical abstract

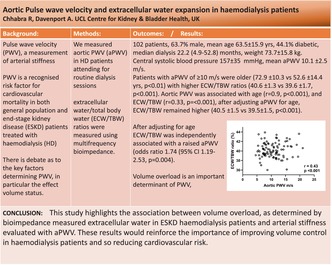

## 1 Background

Despite advances in care, the mortality of end‐stage kidney disease (ESKD) patients remains significantly higher than that of the general population [1], with cardiovascular disease being the major cause of death. Arterial stiffness is a significant risk factor for future cardiovascular events in both the general population and those with ESKD treated with dialysis [2].

Atheromatous arterial disease, characterized by cholesterol‐rich plaque formation, is more commonly found in the general population; however, patients with progressive chronic kidney disease (CKD) and those undergoing dialysis for ESKD typically develop arteriosclerosis, leading to an increased risk of heart failure, stroke, and cardiac arrhythmias [2, 3].

Arteriosclerosis leads to arterial stiffness, and pulse wave velocity (PWV) is the most validated, noninvasive method of determining arterial stiffness, and as such is currently considered to be the gold standard measurement of arterial stiffness [4]. In both the general population and for kidney dialysis patients, aortic stiffness is recognized as a major independent risk factor for cardiovascular death [4, 5, 6].

As CKD progresses then the ratio of small arterial media thickness to lumen diameter increases [7]. Although the mechanistic pathophysiology for this medial hypertrophy and increased arterial stiffness is not fully understood, factors such as chronic fluid overload, mineral bone disorders with vascular calcification, increased vascular smooth muscle sodium content, microinflammation, uremic toxins, and increased visceral sympathetic nervous system overactivity have been implicated. Additionally, the use of higher dialysate calcium concentrations has been reported to increase PWV, whereas middle‐cuff‐off dialyzers and hemodiafiltration may stabilize or reduce PWV [3, 8].

Previous cross‐sectional observational studies of PWV measurements in ESKD hemodialysis patients have varied in their observations, with studies suggesting varying associations with volume overload, inflammation, blood pressure control, choice of dialysate calcium, dialyzer type, and dialysis modality, and equally other studies reporting no such associations [9, 10, 11, 12, 13, 14, 15, 16]. However, one prospective study involving ESKD hemodialysis patients reported that by reducing predialysis extracellular water (ECW) there was an initial reduction in PWV, although this apparent effect on PWV was not sustained, and PWV subsequently increased despite the reduction in ECW [9]. As other studies have not demonstrated an association with ECW [10], we reviewed PWV measurements in a cohort of adult ESKD dialysis patients.

## 2 Patients and Methods

ESKD dialysis patients attending for routine outpatient treatments were recruited into a pilot study measuring aortic pulse wave velocity (aPWV), using the Mobil‐O‐Graph device (IEM, Stolberg, Germany), which has been validated against direct invasive measurements in both children and adults [17]. All measurements were made predialysis with patients lying on a bed in a temperature‐controlled environment. In keeping with standard practice, aPWV measurements were adjusted for heart rate [18], and also recorded as to whether they were higher than expected, greater than the 90% confidence limits for age and gender. All measurements were made by the same observer.

Patients newly starting dialysis, those with recent hospitalizations, individuals unable to undergo bioimpedance measurements (such as those with amputations, paralysis, or immobilized limbs), those unable to provide valid consent due to language barriers or dementia, and those who declined to participate were excluded from this pilot study.

In addition, bioimpedance measurements of extracellular water (ECW), intracellular water (ICW), and body composition were measured by multifrequency bioelectrical impedance assessments (MFBIA) (InBody 720, Seoul, South Korea) on the same day as PWV was recorded, following a standard protocol after patients had emptied the bladder if required [19, 20]. Bioimpedance measurements were made both pre‐ and then postdialysis, after time had been allowed for re‐equilibration postdialysis. Bioimpedance equipment was regularly serviced and calibrated.

Patient demographics, routine laboratory investigations, and relevant medical histories were obtained from computerized medical records, and comorbidity was assessed using the UK Stoke–Davies grading and Charlson comorbidity index without the age factor [21] along with the Clinical Frailty Score (CFS) [22].

Dialysis prescriptions and session details were obtained from computerized records. Patients dialyzed with Fresenius 5008H dialysis machines (Fresenius Medical Company, Bad Homburg, Germany) using high‐flux polysulfone dialyzers and ultrapure‐quality dialysis water, with dialysis sessional urea clearance (Kt/V) calculated using standard equations, and normalized protein appearance rate (nPNA) calculated from pre‐ and post sessional serum urea measurements and 24 h urine collections [23]. All patients dialyzed with dialysate bicarbonate of 28, acetate 3, magnesium 0.5, and glucose 5.5 mmol/L. Dialysate sodium was prescribed by the supervising clinical team, with some 26% prescribed ≤ 136 mmol/L, 40% 137 mmol/L, 30% 138–139 mmol/L, and 4% 140 mmol/L.

All patients were prescribed cholecalciferol 20 000 IU weekly.

Predialysis blood samples were measured in a UK‐accredited laboratory (Roche Integra, Roche Diagnostics, Lewes, UK) for urea, creatinine, albumin, hemoglobin, C‐reactive protein (CRP), glycated hemoglobin (HbA1c), and N‐terminal probrain‐type natriuretic peptide (NT‐proBNP) [24].

Two‐dimensional M‐mode transthoracic echocardiogram (Philips IE33; Philips Medical Systems, Eindhoven, the Netherlands) was performed on a nondialysis day in the left decubitus position by trained and certified cardiology echocardiography technicians after the second or third dialysis session of the week. The American Society of Echocardiography (ASE) recommendations were used to categorize patients according to relative wall thickness (RWT), with ratios of ≤ 0.42 for eccentric and > 0.42 for concentric left ventricular hypertrophy (LVH) respectively [1].

## 3 Ethics

This observational study was approved by the UK National Health Service National Ethics Committee (REC reference: 21/NI/0059), protocol number: 136345, and was undertaken in keeping with the Helsinki Accord, with appropriate written informed consent provided by all participants.

## 4 Statistical Analysis

Data were checked for normality using the D'Agostino–Pearson normality test, and results were expressed as mean ± standard deviation, median, and interquartile range, or percentage. Statistical analysis was by standard tests: for categorical and numerical data using Student's t‐test, Mann–Whitney U test, ANOVA, and Kruskal–Wallis tests, along with Chi square (X2), with appropriate post hoc corrections for multiple testing or small numbers as appropriate. Spearman correlation was used for univariate analysis. Step backward binary logistic regression models were developed for increased aPWV using variables that correlated with changes in aPWV (*p* < 0.1) by univariate analysis. If required, variables were log transformed to improve normality distribution. Variables were then removed or retained in the models if the 95% confidence intervals for the estimate did not include 0 or there was an improvement in model fit (as demonstrated by the –2 log likelihood). Models were checked for collinearity and variable inflation factor to prevent overloading, and variables were checked for collider bias. Log‐rank Mantel–Cox analysis was used for survival analysis. Statistical analysis was performed using GraphPad Prism (version 10.4, GraphPad, San Diego, CA, USA) and SPSS (version 29, IBM, Armonk, New York, USA). Statistical significance was taken at or below the 5% level.

## 5 Results

Data were collected from 102 established ESKD patients attending routine outpatient dialysis sessions, out of a potential 176 patients.

Patient demographics are set out in Table 1. Diabetic kidney disease was the most common cause of ESKD (32.4%), followed by interstitial diseases (24.5%), hypertension (14.7%), glomerulonephritis (12,7%), vasculitis (3.9%), congenital disorders (2.9%), and 8.8% unclassified.

**TABLE 1:** Patient demographics. Patients were divided according to aortic pulse wave velocity (PWV) as < 10 and > 10 m/s, and also after adjustment for age as increased or normal for age and gender. Diabetic (DM), Clinical Frailty Scale (CFS), comorbidity scales (Charlson and Stoke–Davies), dialysis vintage months (vintage mo), prescription of antihypertensive medications (BP meds), number of classes of antihypertensive medications (BP classes), HMG‐CoA reductase inhibitors (statins), active vitamin D_3_ medications (act VitD_3_), elemental calcium in calcium‐containing phosphate binders (Ca mg/day), and patients prescribed cinacalcet (cinacalcet). Data expressed as integer, mean ± standard deviation, median (interquartile range), or percentage.

Variable	All	PWV < 10	PWV > 10	Normal	Increased
Number	102	47	55	35	67
PWV m/s	10.1 ± 2.5	8.2 ± 1.6	11.8 ± 1.8***	8.6 ± 2.1	10.9 ± 2.3***
Male (%)	63.7	70.2	58.2	68.6	61.1
Age years	63.5 ± 15.9	52.6 ± 14.4	72.9 ± 10.3**	60.4 ± 15.4	65.2 ± 16.1
DM (%)	44.1	25.5	60***	31.4	50.7
CFS	4 (3–5)	4 (3–4)	5 (4–6)***	4 (3–5)	4 (3–5)
Charlson	4 (3–5)	3 (3–4)	4 (3–5)**	4 (3–4)	4 (3–5)
Davies	1 (0–2)	1 (0–2)	1 (1–2.5)**	1 (0–2)	1 (0–2)
Vintage mo	22.2 (4.9–52.8)	15.6 (5.8–32.6)	28 (11.2–59.1)*	24.8 (9.6–62.1)	18.5 (6.8–40.8)
% BP meds	81.4	78.7	83.6	80	98.5
BP classes	1 (1–2)	1 (1–2)	1 (1–2)	1 (1–3)	1 (1–2)
Statins (%)	59.5	48.9	69.1	62.9	58.2
Act Vit D_3_ ug/week	2 (0.75–7)	3.5 (0.75–7)	1.75 (0.75–5)	2 (0.75–7)	2 (0.75–6)
Ca mg/day	0 (0–240)	0 (0–180)	60 (0–360)	0 (0–360)	60 (−200)
% Ca meds	48	44.7	50.9	42.9	50.7
% Cinacalcet	11.8	10.6	12.7	10	14.9
Death %	13.7	4.3	21.8*	2.9	19.4*

*Note:* *p < 0.05; **p < 0.01; ***p < 0.001 vs. lower PVW group.

Patients with an aPWV > 10 m/s were older, more likely to be diabetic, with greater comorbidity and frailty scores, and had dialyzed for longer, whereas after age and gender adjustment, demographics were similar (Table 1). There were no significant differences in aPWV and the primary cause of ESKD (data not shown).

Standard biochemical profiles, including lipids and those of mineral bone disorder (MBD), and respective medications, including prescription of active vitamin D_3_ (alfacalcidol), calcium‐containing phosphate binders, and dialysate calcium concentrations, were not different for those with higher aPWV. However, dialysate sodium was higher in those with an aPWV of > 10 m/s, and the gradient between serum and dialysate was lower (Tables 1 and 2). After age and gender adjustment, CRP was higher in those with a higher aPWV, and although there was a trend for a lower serum to dialysate sodium gradient, this was not significant.

**TABLE 2:** Laboratory results and dialysis session prescriptions. Patients were divided according to aortic pulse wave velocity (PWV) as < 10 and > 10 m/s, and also after adjustment for age, as increased or normal for age and gender. Glycated hemoglobin (HbA1c), high‐density lipoprotein (HDL), triglycerides (TG), C‐reactive protein (CRP), parathyroid hormone (PTH), N terminal probrain natriuretic peptide (NT‐proBNP), dialysis sessional urea clearance (Kt/V), normalized nitrogen appearance rate (nPNA), intradialytic weight (IDW), sodium (Na), hemodiafiltration modality (HDF), sodium gradient (serum Na minus dialysate Na), dialysate Na greater than serum Na (negative gradient). Data expressed as integer, mean ± standard deviation, and median interquartile range or percentage.

Variable	All	PWV < 10	PWV > 10	Normal	Increased
Hemoglobin g/L	111.2 ± 13.9	104.6 ± 25	114.6 ± 25**	103.5 ± 28	110.6 ± 18.5
Albumin g/L	39 ± 4.8	39 ± 5	39 ± 5	39 ± 5	39 ± 5
Sodium mmol/L	138 ± 3	139 ± 3	137 ± 3*	139 ± 3	138 ± 3
Calcium mmol/L	2.30 ± 0.2	2.28 ± 0.2	2.36 ± 0.3	2.30 ± 0.3	2.33 ± 0.2
Phosphate mmol/L	1.72 ± 0.5	1.8 ± 0.61	1.64 ± 0.37	1.6 ± 0.47	1.76 ± 0.51
HbA1c mmol/mol	38 (33–47)	36 (32–45)	40 (35–52)	38 (33–47)	38 (33–51)
Cholesterol mmol/L	3.8 ± 1.0	3.9 ± 1.2	3.8 ± 0,9	3.9 ± 1.3	3.8 ± 0.9
HDL mmol/L	1.3 ± 0.5	1.3 ± 0.5	1.4 ± 0.5	1.3 ± 0.4	1.3 ± 0.6
Triglycerides mmol/L	1.3 (0.9–1.7)	1.2 (0.9–1.5)	1.3 (0.8–1.7)	1.3 (1–1.23)	1.2 (0.9–1.6)
HDL/TG ratio	0.97 (0.63–1.51)	0.88 (0.56–1.42)	1.07 (0.7–2.0)	0.88 (0.53–1.5)	1.0 (0.67–1.55)
CRP mg/L	5 (2–13)	6 (2–15)	5 (2–12)	6 (2–24)	5 (2–12)*
PTH pmol/L	36.1 (19.7–63.8)	43.7 (24.7–86.5)	28.5 (17.4–57.2)	36.2 (24.7–90.5)	35.9 (18–54.2)
NT‐proBNP pg/mL	7611 (2551–17 854)	6928 (2156–21 643)	8146 (3242–15 777)	4496 (1867–14 865)	9389 (3461–18 585)
Kt/V	1.18 ± 0.27	1.2 ± 0.3	1.2 ± 0.3	1.2 ± 0.3	1.2 ± 0.3
nPNA g/kg.day	0.83 ± 0.39	0.84 ± 0.34	0.84 ± 0.44	0.81 ± 0.34	0.84 ± 0.42
IDW gain kg	1.65 (1.1–2.1)	1.8 (0.9–2.5)	1.1 (1.0–2.0)	1.8 (0.8–2.2)	1.6 (1.2–2.2)
% IDW gain	2.3 (1.5–2.9)	2.3 (1.2–3.1)	1.6 (1.1–2.8)	2.3 (1.1–2.8)	2.3 (1.6–2.9)
Dialysate sodium mmol/L	137 (136–137)	137 (136–137)	137 (136–138)*	137 (136–138)	137 (136–138)
Dialysate calcium mmol/L	1 (1.0–1.25)	1 (1.0–1.25)	1 (1.0–1.25)	1 (1.0–1.25)	1 (1.0–1.25)
HDF %	92.2	95.7	87.3	88.6	92.5
Convection volume L	15.8 (12.2–20.6)	17 (12.7–21.4)	15.2 (12.2–18.4)	16 (11.9–21)	15.7 (12.4–20.4)
Na gradient mmol/L	1 (−1 to 4)	3 (−1 to 5)	0 (−1 to 2)**	3 (0–4)	0 (−1 to 3)
Negative Na gradient %	47.1	31.9	55.5*	31.4	60.7

*Note:* *p < 0.05;**p < 0.01; ***p < 0.001 vs. lower PVW group.

Patients with an aPWV of > 10 m/s exhibited a different body composition, including lower soft lean mass, appendicular lean mass, and higher body fat. However, after adjusting for age and gender, these differences were no longer significant (Table 3). However, both the ratios of predialysis ECW/ICW and ECW/TBW were greater for patients with higher aPWV. Cardiac imaging was available for review in 96 (94.1%) patients, and most patients had left ventricular hypertrophy, with the majority having concentric left ventricular hypertrophy.

**TABLE 3:** Body composition, and cardiac measurements. Patients were divided according to aortic pulse wave velocity (PWV) as < 10 and > 10 m/s, and also after adjustment for age, as increased or normal for age and gender. Body mass index (BMI), soft lean mass (SLM), appendicular lean mass (ALM), appendicular lean mass index (ALMI), body fat (fat), extracellular water (ECW), intracellular water (ICW), total body water (TBW), central systolic blood pressure (cSBP), diastolic (cDBP), pulse pressure (cPP), cardiac index (CI), left ventricular mass index (LVMI), and ejection fraction (EF%). Data expressed as integer, mean ± standard deviation, median (interquartile range), or percentage.

Variable	All	PWV < 10	PWV > 10	Normal	Increased
Weight kg	73.7 ± 15.8	76.5 ± 16.3	71.4 ± 15.2	75.4 ± 11.2	72.9 ± 14.5
BMI kg/m^2^	26.4 ± 5.0	26.3 ± 5.5	26.4 ± 4.6	26.5 ± 5.5	26.3 ± 4.7
SLM kg	51.9 ± 11.3	56.5 ± 10.5	48.6 ± 10.4***	52.9 ± 11.4	51.4 ± 11.2
ALM kg	21.3 ± 6.1	23.0 ± 5.8	19.6 ± 5.9***	22.2 ± 6.4	20.8 ± 5.9
ALMI kg/m^2^	7.5 ± 1.6	7.9 ± 1.5	7.2 ± 1.6*	7.7 ± 1.5	7.4 ± 1.6
% Fat	29 (19.4–39.8)	22.2 (11.6–32.4)	34.7 (22.9–41.9)**	28.3 (21.5–40.2)	31.9 (17.7–39.7)
ECW/ICW	67.2 ± 4.4	65.8 ± 4.7	68.5 ± 3.7***	65.4 ± 4	68.2 ± 4.3***
ECW/TBW	40.2 ± 1.6	39.6 ± 1.7	40.6 ± 1.3***	39.5 ± 1.4	40.5 ± 1.5***
cSBP mmHg	157 ± 35	149 ± 36	164 ± 34	150 ± 39	160 ± 33
cDBP mmHg	92 ± 20	93 ± 23	92 ± 13	89 ± 21	94 ± 19
cPP mmHg	64.5 ± 25.6	56.0 ± 22.0	72.0 ± 27.0*	61.0 ± 29.0	66.0 ± 24.0
CI L/min.m^2^	2.9 ± 0.6	3.0 ± 0.6	2.8 ± 0.5	2.9 ± 0.6	2.9 ± 0.6
LVMI g/m^2^	107.6 ± 29	108 ± 29	105. ± 29	106 ± 29	107 ± 29
EF %	56.1 ± 8.8	56.0 ± 8.3	56.1 ± 9.2	55.8 ± 9.4	56.3 ± 8.5
LVH%	57.3	60	52.8	54.5	57.8
Concentric%	67.7	60	71.7	55.5	75.0

*Note:* *p < 0.05; **p < 0.01; ***p < 0.001 vs. lower PVW group.

Although diabetic patients had higher aPWV, they were older, with greater clinical frailty and comorbidity scores, along with higher ECW/TBW and ICW/TBW ratios and dialyzed with a greater dialysate to serum sodium gradient (Table S1).

**FIGURE 1:**
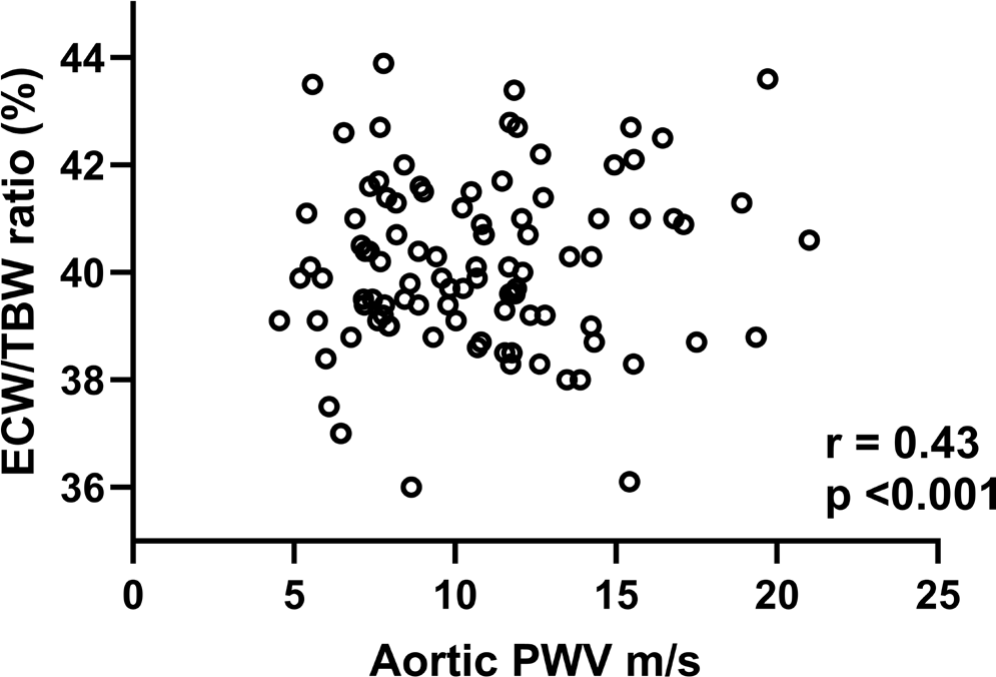
Univariate Spearman association between aortic pulse wave velocity (PWV) adjusted for heart rate and the ratio of extracellular water to total body water (ECW/TBW), expressed as a percentage.

On univariate analysis, aPWV was most strongly associated with age, body composition, hemoglobin, frailty score, predialysis ECW/TBW, ECW/ICW, PTH, left ventricular mass, comorbidity, dialysate to sodium gradient, and dialysis modality (Table 4, Figure 1). Multivariable step backward logistic regression models were analyzed for both aPWV adjusted for heart rate of 70 beats/min and increased aPWV after age and gender adjustment, with variables screened to exclude collider bias. Higher aPWV was independently associated with age and ECW/TBW ratio; after age and gender adjustment, ECW/TBW ratio (Table 5).

**TABLE 4:** Univariate statistically significant associations with pulse wave velocity.

Variable	*r*	*p*
Age years	0.90	< 0.0001
Soft lean mass	−0.40	< 0.0001
Hemoglobin g/L	0.35	0.0003
Clinical frailty score	0.35	0.0003
% Body fat	0.34	0.0005
Parathyroid hormone pg/mL	−0.33	0.0006
Extracellular/intracellular water ratio	0.33	0.0007
Appendicular lean mass	−0.31	0.002
Postdialysis diastolic blood pressure mmHg	−0.29	0.003
Predialysis diastolic blood pressure mmHg	−0.27	0.006
Left ventricular mass g	−0.28	0.007
Charlson comorbidity index	0.25	0.011
Sodium gradient: serum—dialysate mmol/L	−0.25	0.011
Diabetes mellitus	0.25	0.013
Left ventricular end‐diastolic diameter cm	−0.28	0.013
Glycated hemoglobin mmol/mol	0.30	0.034
Stoke–Davies comorbidity	0.21	0.037
HMG‐CoA reductase prescription	0.21	0.037
Hemodiafiltration mode	−0.20	0.043

**TABLE 5:** Multivariable step backward models of variables independently associated with an increased pulse wave velocity > 10 m/s, and above reference range for age and gender. Ratio of extracellular water (ECW) to total body water (TBW), soft lean mass (SLM), hemoglobin (Hb), standard error of β (StE β), odds ratio (OR), and 95% confidence interval (95% CI). Models Nagelkerke *r* ^*2*^ 0.60 and 0.18 respectively.

Variable	*β*	StE β	Wald	OR	95% CI	*p*
PWV > 10 m/s						
Age year	0.13	0.28	20.1	1.13	1.07–1.96	< 0.001
ECW/TBW	0.47	0.21	5.0	1.59	1.06–2.41	0.026
SLM kg	−0.08	0.04	3.8	0.92	0.84–1.00	0.052
Age‐adjusted increased PWV						
ECW/TBW	0.55	0.19	8.2	1.74	1.19–2.53	0.004
Hb g/L	0.03	0.02	3.1	1.03	0.99–1.06	0.077

Patients recruited between December 2022 and December 2023 were followed until the end of December 2024, median of 18.8 months (15–24.9), and mortality was greater for those with higher aPWV (Table 1), and by Kaplan–Meier analysis X2 = 2.4, *p* = 0.029, for aPWV > 10 m/s and after age and gender adjustment, respectively (*p* = 0.028).

## 6 Discussion

Aortic PWV is a measure of vascular stiffness, which can be influenced by several factors. These include internal forces generated by the volume of blood and viscosity, the stiffness of the vascular wall caused by vascular calcification and nonosmotic sodium accumulation in vascular smooth muscle, and tissue matrix proteoglycans. Additionally, balances of local vasodilators and vasoconstrictors, visceral sympathetic stimulation, and the use of antihypertensive medications also contribute to arterial stiffness [1, 2, 3].

Arterial stiffness generally increases with age and uncontrolled hypertension [5, 18], but there has been debate as to the role of volume overload compared to vascular calcification in patients with ESKD treated by dialysis [3, 9, 14]. Several previous small cross‐sectional studies did not observe an association between volume overload, as assessed by bioimpedance, and PWV [25, 26]. However, there were age differences between the fluid‐overloaded and normovolemic groups. In this study, we report an association between volume overload and increased aPWV, which remained significant even after adjusting for age and gender. This would support an earlier longitudinal study that reported that PWV decreased following a reduction in postdialysis target weight and fluid overload as assessed by lung ultrasound [27].

As observed in many previous studies, patients with increased aPWV had increased mortality, even after adjusting for age [2, 6, 11]. Similarly, our cohort of patients, with diabetic nephropathy as the primary cause of ESKD, had higher aPWV, in keeping with previous reports [3]. Although our diabetic patients were older and had greater comorbidity, they were also classified as more volume overloaded by bioimpedance measurements.

Previous reports have also reported that ESKD patients secondary to hypertensive kidney disease as the primary cause of ESKD have increased PWV [6]; however, we were unable to confirm this in our study, possibly due to the relatively small sample size.

There has been ongoing debate as to whether calcium balance during dialysis and the choice of dialysate calcium affect PWV [14, 15]. In contrast to many other studies, our cohort was dialyzed using lower dialysate calcium concentrations, and only a minority were prescribed calcium‐containing medications. We observed an association between higher aPWV and lower parathyroid hormone (PTH) concentrations. Lower PTH levels are associated with reduced bone turnover, which in turn increases the risk of vascular calcification in dialysis patients [28]. However, in this cross‐sectional study, vascular calcification was not directly measured, and PTH was not retained as a significant variable in the statistical models.

On the other hand, aPWV was associated with both ECW/TBW ratios and higher dialysate sodium to serum gradient, suggesting that volume overload and less diffusive sodium clearance with dialysis may potentially contribute to an increased aPWV [29]. On multivariable analysis, ECW/TBW was independently associated with aPWV. Normal aging can lead to changes in body composition with loss of lean body mass and gain in fat mass, which could increase the ECW/TBW ratio [30]. However, after adjusting aPWV for age, ECW/TBW remained independently associated with aPWV, suggesting a potential role for volume expansion in increasing aPWV [3]. As such, our results would support those of two previous longitudinal studies, which reported a decrease in PWV following a reduction in ECW, as assessed by bioimpedance and lung ultrasound, respectively [9, 27]. In addition to volume status, blood viscosity also affects the internal pressure exerted on the arterial wall. Hemoglobin concentration, a primary determinant of blood viscosity, was retained in the model, although its association with aPWV did not reach statistical significance.

As with all observational studies, we can only report associations. Our results must be interpreted in the context of the specific patient population when compared to previous studies. Our patients dialyzed with markedly lower calcium dialysate concentrations, and only a minority were prescribed calcium‐containing medications. Additionally, vascular calcification was not measured as part of our study. Dietary intake was assessed solely by nPCR, and we did not collect detailed dietary histories of calcium or sodium intake. It must be recognized that bioimpedance can be influenced by changes in body composition, as muscle has a very high water content, while fat has a much lower content. Thus, changes in body composition can influence bioimpedance assessments of volume overload, in particular, the ratio of ECW/TBW [31]. However, when we compared patients after adjusting aPWV for the effect of age, there were no significant differences in estimates of body composition.

In contrast to previous studies which measured PWV between the aorta and various conduit arteries, we chose to measure aPWV. This method was selected to reduce potential errors related to the measurement of pulse wave travel distance, differences in PWV between the aorta and conduit arteries, and the varying propensity for developing atheroma and vascular calcification across different arterial sites [4, 18].

Although PWV is a well‐recognized risk factor for cardiovascular mortality in ESKD dialysis patients, there is ongoing debate as to its main determinants and thus potential therapeutic strategies to prevent progression and reduce mortality risk. Given that aPWV does not necessarily increase over time in ESKD dialysis patients [10, 13], this suggests that potentially reversible factors, such as volume overload, are important in determining aPWV. The results of this study suggest a potential role for volume overload leading to an increase in aPWV, and that greater attention should be given to ensure that hemodialysis patients are encouraged to limit interdialysis weight gains and achieve postdialysis target weights. However, further studies would be needed to investigate whether sodium balance [32] underlies the changes in volume status and PWV.

## Author Contributions

R.C. conducted the study. A.D. obtained ethical approval. Both authors analyzed data and approved the submitted manuscripts.

## Conflicts of Interest

The authors declare no conflicts of interest.

## Supporting information


Data S1.
