# Supplementary material for: Aortic Pulse Wave Velocity and Extracellular Water Expansion in Hemodialysis Patients
Source: Artif Organs. 2025 May 7;49(9):1429–36. doi: 10.1111/aor.15015 (PMC12548306; doi:10.1111/aor.15015)
Supplement: Supplementary file 1 — Data S1. [file AOR-49-1429-s001.doc]

Supplementary table

Table 1. Patients divided according to diagnosis of diabetes mellitus. Only variables with a statistical difference are reported. HMG-CoA reductase inhibitors (statins), sodium (Na). Data expressed as integer, mean ± standard deviation, median (interquartile range) or percentage. * p<0.05, ** p<0.01, *** p<0.001 vs non-diabetic group.

| variable | Non-diabetic | diabetic |
| --- | --- | --- |
| number | 57 | 45 |
| Pulse wave velocity m/s | 9.5 ±2.5 | 10.9 ±2.4** |
| Pulse wave velocity >10 m/s | 38.6 | 73.3*** |
| Age years | 60.8±15.8 | 67.2±15.5* |
| Clinical Frailty Score | 4 (3-5) | 4 (4-6)** |
| Charlson comorbidity index | 3 (3-4) | 5 (4-5)*** |
| Statins (%) | 49.1 | 73.3* |
| Active Vitamin D_3_ ug/week | 5 (01.75-7) | 1.5(0.75-2.0)** |
| Phosphate mmol/L | 1.83±0.55 | 1.57±0.37* |
| Albumin g/L | 39.9±4.4 | 37.1±4.8** |
| Cholesterol mmol/L | 4.0±1.1 | 3.6±0.9* |
| Glycated haemoglobin mmol/mol | 34 (31-35) | 47 (41-60)*** |
| Extra/Intra-cellular water %ratio | 65.4 ±3.7 | 69,5 ±4.1*** |
| Extracellular/Total Body water %ratio | 39.5 ±1.4 | 41.0 ±1.4 *** |
| Dialysate sodium mmol/L | 137 (136-137) | 137 (137-137)* |
| Serum-Dialysate Na gradient mmol/L | 2 (0-4) | 0 (-1 to 3)* |
| Dialysate sodium > Serum sodium % | 21.1 | 42.2 * |
| Death % | 5.3 | 24.4** |
